# Evaluation of Semicircular Canal Function Using the Video Head Impulse Test in a Case of Relapsing Polychondritis and Otitis Media With Effusion

**DOI:** 10.7759/cureus.92184

**Published:** 2025-09-12

**Authors:** Keishi Fujiwara, Shinya Morita, Hideaki Takeda, Akihiro Homma

**Affiliations:** 1 Department of Otolaryngology - Head and Neck Surgery, Faculty of Medicine, Graduate School of Medicine, Hokkaido University, Sapporo, JPN

**Keywords:** caloric testing, otitis media with effusion, relapsing polychondritis, semicircular canal function, video head impulse test

## Abstract

Relapsing polychondritis (RP) is a rare, immune-mediated disorder characterized by recurrent inflammation of cartilaginous structures, often affecting the ears, nose, and respiratory tract. Vestibular dysfunction in RP is uncommon but clinically significant, resulting from inner ear involvement. We describe a case of an 81-year-old man with RP who presented with fever, bilateral hearing loss, vertigo, and ocular inflammation. Bilateral otitis media with effusion (OME) was identified on imaging and otoscopy, precluding the use of caloric testing. Video head impulse test (vHIT) revealed bilateral semicircular canal dysfunction, with decreased vestibulo-ocular reflex gains and catch-up saccades. The patient was treated with corticosteroid pulse therapy, oral prednisolone, azathioprine, and bilateral myringotomies. While hearing thresholds improved significantly following treatment, vestibular function remained impaired on serial vHIT assessments conducted before and after therapy. This case highlights the utility of vHIT in assessing vestibular dysfunction in RP, especially when middle ear pathology renders traditional caloric testing unreliable. Compared to caloric testing, vHIT offers a minimally invasive, repeatable, and well-tolerated method for evaluating high-frequency vestibular function. Incorporating vHIT into the diagnostic and follow-up process may facilitate earlier recognition of vestibular involvement in RP and provide a useful tool for monitoring treatment response. Given that vestibular dysfunction may not improve in parallel with hearing recovery, early detection and longitudinal evaluation are essential for comprehensive patient care.

## Introduction

Relapsing polychondritis (RP) is a rare, immune-mediated systemic disorder characterized by recurrent inflammation of cartilaginous tissues throughout the body. The disease typically affects the ears, nose, and larynx/trachea, causing visible changes such as redness, swelling, and deformity. In addition to these more obvious manifestations, RP can also involve the inner ear, leading to sensorineural hearing loss and vestibular symptoms such as vertigo, which may significantly impact balance and daily activities [1]. Because the onset and severity of symptoms can vary widely among patients, early recognition and monitoring are important for preventing long-term complications. Additionally, inflammation of the cartilage surrounding the Eustachian tube may impair its function, resulting in otitis media with effusion (OME) [2]. This middle ear condition presents a particular challenge when evaluating vestibular function, as the presence of effusion can interfere with caloric testing [3]. Although air-caloric testing may still be feasible in patients with tympanic membrane perforation following myringotomy, water-caloric testing should be avoided due to potential complications. The video head impulse test (vHIT) is a more recent diagnostic tool that enables assessment of semicircular canal function at high frequencies and under near-physiological conditions [4]. Unlike caloric testing, vHIT is not significantly influenced by middle ear pathology and can therefore be applied even in cases of OME [3,5]. Here, we report a rare case of RP presenting with vertigo and bilateral OME, in which vHIT enabled multiple evaluation of semicircular canal function before and after treatment, providing valuable insights into disease-related vestibular involvement and therapeutic response.

## Case presentation

An 81-year-old man presented with fever and cough. He was initially treated with antibiotics under the presumptive diagnosis of bacterial pneumonia; however, his fever persisted and inflammatory markers continued to worsen. Ten days after the onset of fever, he developed conjunctival hemorrhage and was diagnosed with uveitis by an ophthalmologist. Swelling of the right auricular cartilage appeared another 10 days later, raising suspicion for relapsing polychondritis (RP), and he was admitted to our rheumatology department for further evaluation and treatment. Subsequently, approximately two weeks after the auricular swelling, he experienced rapidly progressive bilateral hearing loss and vertigo. He was referred to our department for further investigation, including auricular cartilage biopsy and assessment for inner ear involvement.

**Figure 1:**
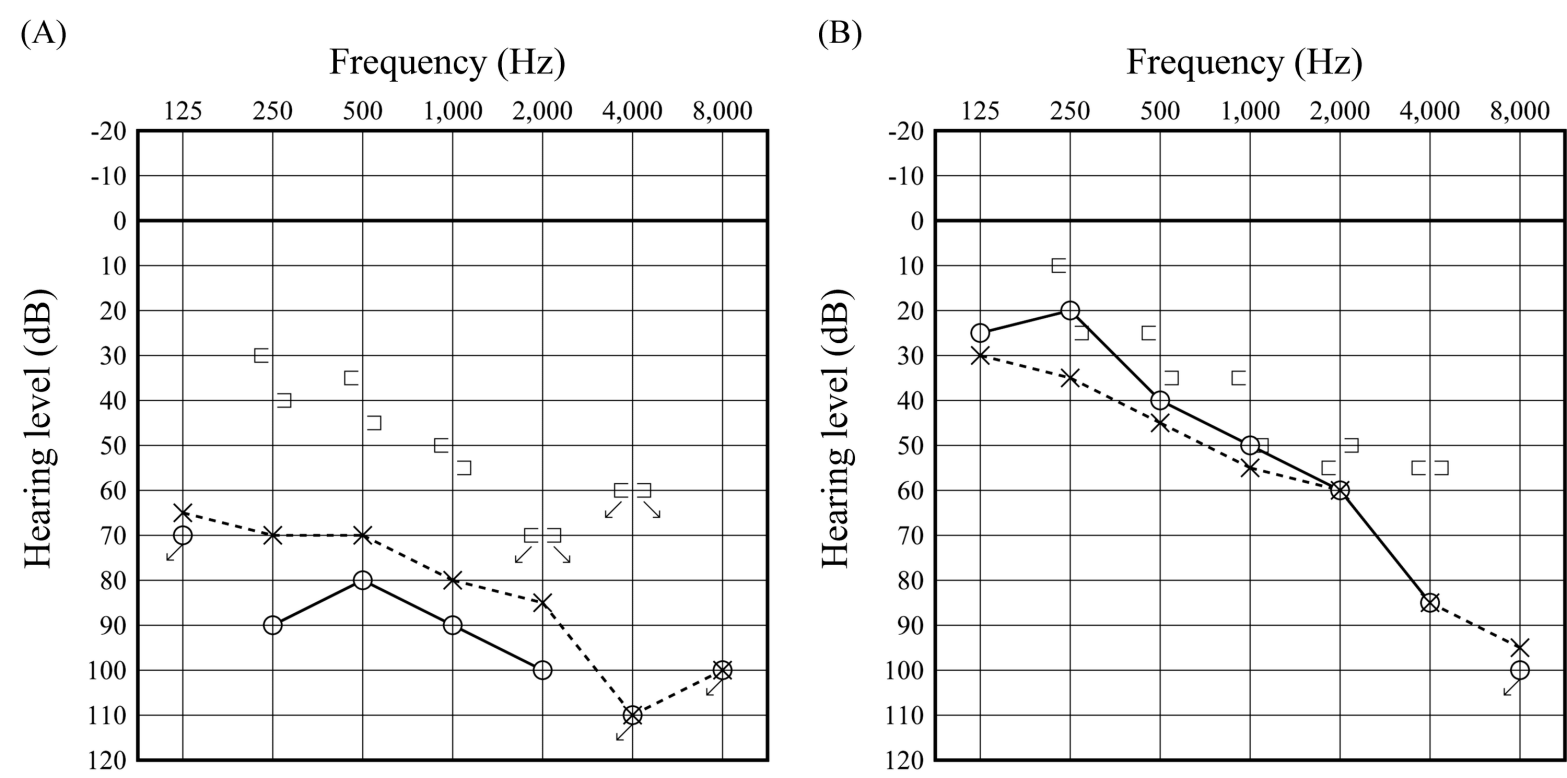
Pure-tone audiograms. Audiograms obtained before treatment (A) and after treatment (B). The pre-treatment PTA demonstrated bilateral mixed hearing loss, with thresholds of 90.0 dB on the right ear and 78.8 dB on the left ear. The post-treatment PTA showed thresholds of 50.0 dB on the right ear and 53.8 dB on the left ear, along with resolution of the air-bone gap. The average was obtained by summing the hearing thresholds at 500 Hz, twice at 1000 Hz, and 2000 Hz, and then dividing the total by four: PTA = (500 Hz + 2 × 1000 Hz + 2000 Hz)/4.

Physical examination revealed redness and swelling of the right auricular cartilage, as well as redness of the left external auditory canal. Both tympanic membranes appeared thickened. There was no tenderness over the nasal, laryngeal, or tracheal cartilage, and fiberoptic laryngoscopy showed no evidence of inflammation. Pure-tone audiometry demonstrated bilateral mixed hearing loss, with thresholds of 90.0 dB on the right and 78.8 dB on the left (Figure 1, panel A). The average was obtained by summing the hearing thresholds at 500 Hz, twice at 1000 Hz, and at 2000 Hz, and dividing the total by four: $\text{PTA} = \frac{\text{Threshold}_{500\,\text{Hz}} + 2 \times \text{Threshold}_{1000\,\text{Hz}} + \text{Threshold}_{2000\,\text{Hz}}}{4}$.

**Figure 2:**
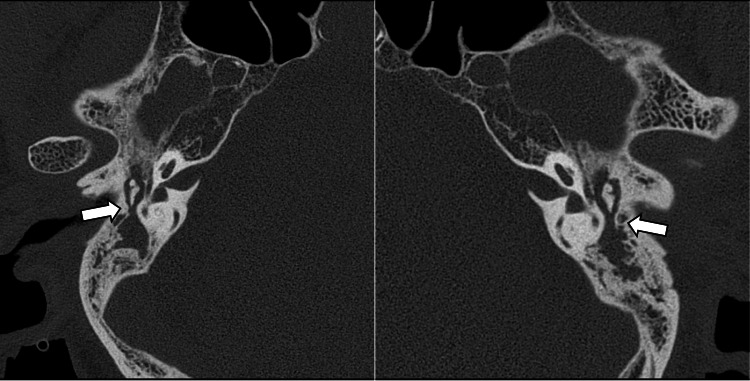
Axial computed tomography (CT) scan of the temporal bones. Bilateral mastoid hypopneumatization and soft tissue densities extending from the mastoid cavities into the tympanic cavities were observed (arrows).

**Figure 3:**
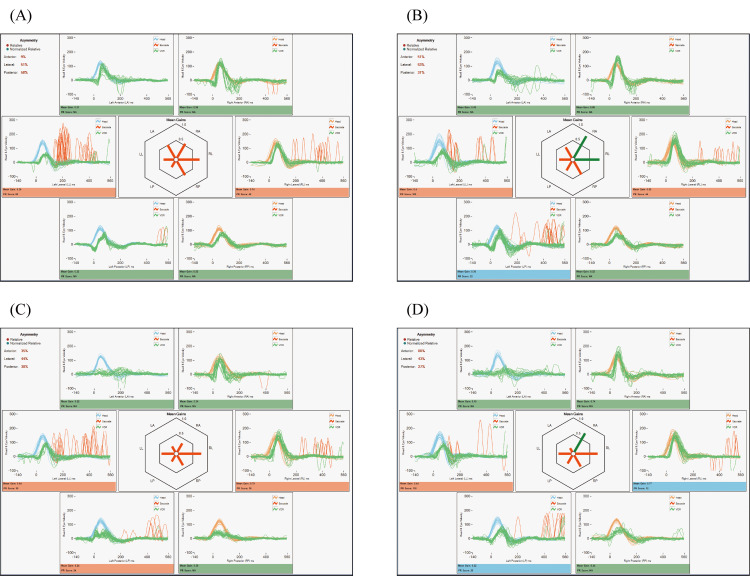
Video head impulse test (vHIT) findings. vHIT results at pre-treatment (A), one week (B), two weeks (C), and one month (D) after treatment. At pre-treatment, bilateral horizontal semicircular canal dysfunction was detected. Although the number of catch-up saccades decreased, no significant improvement in vestibulo-ocular reflex (VOR) gain was observed across the four serial vHIT assessments.

Given the rapid progression of hearing loss and the fact that the bone conduction thresholds were worse than the age-specific average hearing levels, we considered that the hearing loss was not due to presbycusis. Temporal bone computed tomography (CT) showed bilateral mastoid hypopneumatization and soft tissue densities extending from the mastoids into the tympanic cavities (Figure 2). No spontaneous nystagmus was observed under infrared charge-coupled device camera monitoring. However, a rightward horizontal nystagmus was elicited following head-shaking, and vHIT demonstrated decreased vestibulo-ocular reflex (VOR) gain predominantly on the left, accompanied by catch-up saccades. These findings indicated bilateral semicircular canal dysfunction (Figure 3, panel A). Caloric testing was not performed due to the presence of middle ear effusion. No responses were elicited on cervical and ocular vestibular evoked myogenic potentials (cVEMP and oVEMP) bilaterally, even with bone-conducted stimuli. Eye tracking tests and optokinetic nystagmus assessments were normal, with no findings suggestive of central vestibular dysfunction. Biopsy of the right auricular cartilage revealed only mild lymphocytic infiltration in the perichondrial connective tissue. There were no histopathological features typical of RP, such as eosinophilic degeneration or cartilage destruction. Bone marrow biopsy was performed to rule out vacuoles, E1 enzyme, X-linked, autoinflammatory, somatic (VEXAS) syndrome; however, no signs of myelodysplastic syndrome (MDS) were found, and VEXAS was considered unlikely. At the initial visit to our department, the white blood cell count was 6,800/µL, which was within the normal range, whereas the C-reactive protein (CRP) level was elevated at 8.08 mg/dL. Antinuclear antibody (ANA) testing was negative. Both myeloperoxidase (MPO) and proteinase 3 (PR3)-antineutrophil cytoplasmic antibody (ANCA) were negative.

Given the presence of auricular chondritis, uveitis, and cochleovestibular dysfunction, a diagnosis of RP was made based on Damiani’s criteria [6]. The patient was also diagnosed with bilateral OME. Treatment was initiated with methylprednisolone pulse therapy (1000 mg/day for three days), followed by tapering oral prednisolone starting at 60 mg/day and concomitant initiation of azathioprine. Bilateral myringotomies were performed, resulting in drainage of serous effusion and improvement in air conduction thresholds in both ears. Bone conduction thresholds also gradually improved, with final audiometric testing showing thresholds of 50.0 dB on the right and 53.8 dB on the left, and resolution of the air-bone gap (Figure 1, panel B). The patient reported subjective improvement in balance, although no significant improvement in semicircular canal function was observed across four serial vHIT assessments conducted before and after treatment (Figure 3, panels B-D). He was transferred to a local hospital for continued care when the prednisolone dose had been tapered to 30 mg/day.

## Discussion

In the present case of RP with concurrent OME, serial evaluations of semicircular canal function were performed using vHIT before and after treatment. The utility of vHIT in assessing semicircular canal dysfunction in RP has been previously reported [7]. This study evaluated three RP patients with dizziness or imbalance using vHIT and demonstrated bilateral semicircular canal hypofunction in all cases, with a significant correlation between the degree of vestibular dysfunction and sensorineural hearing loss [7]. In the current case, semicircular canal function could not be adequately assessed by caloric testing due to the presence of OME, further highlighting the clinical value of vHIT. Moreover, since bilateral myringotomy had been performed and perforations remained in both tympanic membranes, vHIT proved to be a useful method for evaluating vestibular function under such conditions.

Although the incidence of OME in RP is relatively low, the inclusion of vestibular dysfunction as one of the six clinical features in McAdam's diagnostic criteria underscores the importance of vestibular assessment in the diagnostic process [8]. In this context, vHIT represents a practical and informative tool for evaluating semicircular canal function. Compared to caloric testing, vHIT is less likely to provoke unpleasant symptoms such as nausea, making it more suitable for repeated testing and for monitoring changes in vestibular function throughout the course of treatment. While vHIT provides high-frequency vestibular assessment and is less affected by middle ear pathology, it may overlook low-frequency deficits detectable by caloric testing.

Vestibular dysfunction is reported in approximately 20% of RP cases, yet literature specifically addressing balance dysfunction in RP remains limited [9]. While some case reports have documented bilateral vestibular hypofunction based on caloric testing [10], others have utilized vHIT to detect semicircular canal dysfunction [11]. In the present case, steroid therapy was initiated relatively early, approximately one month after symptom onset; however, no significant improvement in semicircular canal function was observed on repeated VHIT assessments. Similarly, Hoshino et al. reported that vestibular dysfunction in RP did not improve with treatment [7]. In contrast, early steroid intervention has been shown to improve hearing outcomes in RP-related sensorineural hearing loss [12]. Whether semicircular canal function, like hearing, can improve with early treatment remains uncertain; it is also possible that vestibular dysfunction is less responsive to therapy than auditory dysfunction. Further case data accumulation will be necessary to clarify this issue.

In this case, the patient exhibited multiorgan inflammatory involvement including auricular chondritis, ocular inflammation, hearing loss, and vertigo. Given this clinical presentation, vacuoles, E1 enzyme, X-linked, autoinflammatory, somatic (VEXAS) syndrome was included in the differential diagnosis. VEXAS syndrome is an adult-onset autoinflammatory disease that predominantly affects older men and is characterized by systemic inflammation, skin rash, chondritis, pulmonary involvement, vasculitis, fever, and cytopenias, often in association with hematologic disorders such as MDS [13]. There is substantial clinical overlap between RP and VEXAS, particularly in elderly men presenting with auricular chondritis, ocular symptoms, and systemic inflammation. In the present case, bone marrow examination was performed to rule out VEXAS syndrome, but no morphological abnormalities or dysplasia suggestive of MDS were observed, making the diagnosis of VEXAS unlikely. Nevertheless, in patients diagnosed with RP - especially those with treatment resistance or hematological abnormalities - evaluation for VEXAS syndrome remains essential.

RP is notoriously difficult to diagnose, with a reported average interval of 2.9 years from symptom onset to definitive diagnosis [14]. Since vestibular dysfunction is included among the diagnostic features of RP, early detection of semicircular canal hypofunction using vHIT may facilitate earlier diagnosis and treatment. Given its minimal patient burden and low likelihood of inducing nausea compared to caloric testing, vHIT should be considered a frontline vestibular assessment in suspected RP. Furthermore, its suitability for repeated testing allows for serial monitoring of treatment effects. In the present case, multiple vHIT evaluations before and after treatment enabled objective assessment of vestibular function and treatment efficacy.

## Conclusions

vHIT is a valuable tool for evaluating vestibular function in patients with RP, especially in the presence of OME, where caloric testing is not feasible. In the present case, serial vHIT assessments provided objective evidence of bilateral semicircular canal dysfunction, although vestibular function did not recover with immunosuppressive treatment, in contrast to the recovery of hearing. These findings suggest that vestibular damage in RP may be less responsive to therapy and potentially irreversible. vHIT should be considered as a frontline vestibular assessment in RP patients presenting with dizziness or imbalance, as it enables early diagnosis and facilitates longitudinal monitoring of treatment effects.
